# ACOT1-specific expression modulates metabolic reprogramming in diabetic cardiomyopathy: The role of SREBP1c lactylation in CD36-mediated lipotoxicity

**DOI:** 10.1016/j.ahjo.2026.100809

**Published:** 2026-06-06

**Authors:** Zeyu Liao, Yahui Fu, Ran Li

**Affiliations:** a The First Affiliated Hospital of Zhengzhou University, Zhengzhou, China; b People's Hospital of Zhengzhou, Zhengzhou, China

**Keywords:** Diabetic cardiomyopathy, ACOT1, SREBP1c, Lactylation, Metabolic reprogramming, Cardiac dysfunction

## Abstract

**Objective:**

Diabetic cardiomyopathy (DCM) is characterized by metabolic dysfunction and lipotoxicity. The roles of acyl-CoA thioesterase 1 (ACOT1) and the novel post-translational modification lactylation in its pathogenesis remain unclear. This study aimed to investigate the stage-specific function of ACOT1 and the mechanism by which lactylation of SREBP1c regulates lipid metabolism in DCM.

**Methods:**

Key genes were screened via bioinformatic analysis. ACOT1 was functionally assessed in early and decompensated DCM mouse models using gain- and loss-of-function strategies, evaluated by echocardiography and hemodynamics. SREBP1c lactylation was identified by liquid chromatography-tandem mass spectrometry (LC-MS/MS) and validated via site-directed mutagenesis.

**Results:**

ACOT1 exhibited a biphasic expression pattern: protective upregulation in early DCM improved cardiac function and attenuated oxidative stress, whereas its downregulation in decompensated DCM exacerbated remodeling and dysfunction. Mechanistically, ACOT1 physically interacted with SREBP1c and facilitated its lactylation under high-lactate conditions. This modification was essential for SREBP1c transcriptional activity, driving its nuclear translocation and subsequent upregulation of the fatty acid transporter CD36. The enhanced CD36-mediated uptake led to free fatty acid accumulation, aggravating myocardial lipotoxicity and DCM progression.

**Conclusion:**

This study reveals a dual-stage regulatory role of ACOT1 in DCM and identifies a novel ACOT1-SREBP1c(lactylation)-CD36 axis linking metabolic reprogramming to lipotoxic injury. These findings establish lactylation as a key regulatory mechanism in diabetic heart metabolism and propose ACOT1 and SREBP1c lactylation as potential therapeutic targets for mitigating myocardial lipotoxicity in DCM.

## 1 Background

The global prevalence of diabetes mellitus (DM) has been rising exponentially over the past few decades, imposing a heavy burden on public health systems worldwide [1], [2]. Diabetic cardiomyopathy (DCM), a specific myocardial pathological change induced by chronic hyperglycemia and metabolic disorders, is recognized as an independent risk factor for heart failure (HF) and cardiovascular mortality in diabetic patients, which is not secondary to hypertension, coronary artery disease, or other traditional cardiovascular comorbidities [3], [4]. The pathological progression of DCM is characterized by a series of structural and functional abnormalities, including myocardial hypertrophy, interstitial fibrosis, mitochondrial dysfunction, and impaired cardiac systolic/diastolic function. Among these pathological features, metabolic reprogramming—especially aberrant lipid metabolism—serves as the initiating and core driver of DCM development, yet the underlying molecular mechanisms remain to be fully elucidated.

Lipid metabolism disorder is a hallmark of DCM, manifested by excessive accumulation of free fatty acids (FFAs), triglycerides, and lipid intermediates in myocardial cells, which triggers myocardial lipotoxicity, oxidative stress, and inflammatory responses, ultimately leading to cardiomyocyte injury and cardiac dysfunction. Unsaturated fatty acids, as essential components of myocardial lipid metabolism, play dual roles in maintaining cardiac homeostasis and inducing pathological damage: physiological levels of unsaturated fatty acids participate in membrane synthesis and energy supply, while their metabolic disorder disrupts lipid homeostasis and accelerates DCM progression. Currently, the regulatory network governing unsaturated fatty acid metabolism in DCM is not fully understood, and identifying key regulatory genes in this pathway is crucial for unraveling DCM pathogenesis [5], [6].

Acyl-CoA thioesterase 1 (ACOT1), a member of the acyl-CoA thioesterase family, is a key regulator of fatty acid homeostasis [7]. It exerts its biological function by hydrolyzing long-chain acyl-CoA esters into free fatty acids and CoA, thereby modulating the balance between fatty acid synthesis, oxidation, and storage [8], [9]. ACOT1 is widely expressed in metabolic tissues such as the liver, adipose tissue, and heart, and its dysregulation has been implicated in multiple metabolic diseases, including obesity, non-alcoholic fatty liver disease, and atherosclerosis [10]. Emerging evidence suggests that acyl-CoA family proteins are closely associated with cardiovascular disease progression: for instance, acyl-CoA binding protein (ACBP) promotes DCM development by enhancing myocardial lipid accumulation, while ACOT7 alleviates cardiac hypertrophy via regulating fatty acid oxidation [8], [11]. However, the expression pattern, stage-specific regulatory role, and downstream molecular targets of ACOT1 in DCM remain largely unclear [12]. Notably, the dual-stage (early protective vs. decompensated pathogenic) expression feature of ACOT1 in DCM, as suggested by our preliminary data, further highlights the need to clarify its functional complexity in DCM progression [13], [14].

Post-translational modifications (PTMs) are crucial for regulating protein function and signaling transduction, and their dysregulation is closely linked to the pathogenesis of various cardiovascular diseases [15], [16]. Lactylation, a novel evolutionarily conserved PTM discovered in 2019, has emerged as a frontier hotspot in the intersection of metabolism and epigenetics [17]. Unlike other PTMs, lactylation is a metabolite-derived modification that directly senses intracellular lactate levels—an end product of glycolysis—and dynamically regulates protein activity, stability, subcellular localization, and transcriptional function [16], [18]. Lactate levels are significantly elevated in DCM due to enhanced glycolysis and impaired mitochondrial metabolism, making lactylation a potential key regulatory mechanism in DCM [19], [20]. Recent studies have confirmed that lactylation participates in the regulation of cardiovascular pathophysiological processes, such as myocardial fibrosis, ischemia-reperfusion injury, and heart failure: for example, lactylation of Snail promotes myocardial fibrosis by enhancing its transcriptional activity, while lactylation of α-myosin heavy chain (α-MHC) impairs cardiac contractile function [21], [22], [23]. Additionally, the first lactylation modification profile of diabetic myocardial infarction has been established, revealing that lactylation extensively modulates lipid metabolism-related proteins, which hints at its potential regulatory role in DCM lipid metabolism [21], [24].

Sterol regulatory element-binding protein 1c (SREBP1c) is a master transcription factor governing lipid metabolism, which directly regulates the expression of genes involved in fatty acid synthesis, uptake, and storage [25], [26]. Aberrant activation of SREBP1c is a key driver of myocardial lipotoxicity in DCM, and targeting SREBP1c has been considered a potential therapeutic strategy for DCM [27], [28]. However, whether SREBP1c can be regulated by lactylation, and how this modification modulates its function in DCM, have not been reported. Given that ACOT1 regulates lipid metabolism and lactate levels are elevated in DCM, we hypothesize that ACOT1 may modulate SREBP1c function via regulating its lactylation, thereby affecting lipid metabolism and DCM progression [28], [29].

In summary, DCM is a devastating complication of diabetes with unclear pathogenesis and limited therapeutic options. The stage-specific role of ACOT1 in DCM, the regulatory effect of lactylation on SREBP1c, and the crosstalk between these molecules in lipid metabolism remain unaddressed. Therefore, this study aims to explore the regulatory mechanism of the ACOT1-SREBP1c (lactylation)-CD36 axis in DCM, clarify the dual-stage function of ACOT1 and the role of SREBP1c lactylation in DCM progression, and provide new insights into the pathological mechanisms of DCM, as well as potential therapeutic targets for clinical intervention.

## 2 Materials and methods

### 2.1 Mouse model

Male C57BL/6 N mice (8 weeks old) were obtained from Huachuang Sino (Taizhou, China) and maintained under standard laboratory conditions (22 ± 1 °C, 45–55% humidity, 12 h light/dark cycle) with free access to food and water. All animal procedures were performed in accordance with the institutional guidelines and approved by the Experimental Animal Ethics Committee of Zhengzhou University Medical School.

To induce type 2 diabetes mellitus (T2DM), age-matched mice were administered a single intraperitoneal injection of streptozotocin (STZ, 120 mg/kg dissolved in citrate buffer, pH 4.5). Following injection, mice were maintained on a standard chow diet until 16 weeks of age. Body weight and fasting blood glucose (FBG) levels were monitored weekly. For FBG measurement, mice were fasted for 12 h (9:00 PM to 9:00 AM), and blood glucose was determined using a portable glucometer (Sannuo, Changsha, China). Mice exhibiting FBG levels persistently >11.1 mmol/L on two consecutive weekly measurements were considered diabetic and included in the study. A cohort of diabetic mice (*n* = 6 per group) subsequently underwent transthoracic echocardiography. Mice exhibiting characteristic echocardiographic features of dilated cardiomyopathy, such as left ventricular chamber dilation and reduced ejection fraction, were confirmed as having diabetic cardiomyopathy (DCM) and used for subsequent interventions.

### 2.2 Longitudinal natural history cohort

A separate cohort of HFD/STZ-induced diabetic mice (*n* = 5 per time point) was maintained without AAV intervention for longitudinal profiling of metabolic, echocardiographic, and histopathological parameters. Mice were sacrificed at weeks 16, 17, 18, 19, 20, 21, and 22 for tissue and blood collection. This cohort was used exclusively to define disease stages and characterize the natural progression of metabolic and functional parameters (Supplementary Table S1), and was distinct from the cohorts used for AAV-mediated ACOT1 gain- and loss-of-function experiments.

### 2.3 Adeno-associated virus and viral delivery protocol

For cardiac-specific knockdown of ACOT1, we generated recombinant adeno-associated virus serotype 9 (AAV9) vectors driven by the cardiac troponin T (cTnT) promoter. Two constructs were packaged: AAV9-cTnT-shACOT1 expressing a short hairpin RNA targeting mouse Acot1, and AAV9-cTnT-FLAG expressing a FLAG-tagged control sequence. One week after the establishment of the diabetic cardiomyopathy model, mice were randomly assigned to groups and injected via the tail vein with 1 × 10^11^ viral genomes of the respective AAV9 vectors in sterile phosphate-buffered saline.Six weeks following surgery, cardiac function was assessed in all mice by transthoracic echocardiography under light isoflurane anesthesia. Subsequently, mice were euthanized by cervical dislocation under deep anesthesia. Heart tissues were promptly excised, rinsed in cold PBS, blotted dry, weighed, and photographed. Ventricular tissues were either snap-frozen in liquid nitrogen for molecular analyses or fixed in 4% paraformaldehyde for histological examination. Blood samples were collected via cardiac puncture, and serum was separated by centrifugation and stored at −80 °C for further biochemical assays.

### 2.4 Echocardiographic assessment

Cardiac systolic and diastolic function was evaluated by transthoracic echocardiography using a VINNO 6 LAB ultrasound imaging system equipped with a 30 MHz linear transducer. Mice were anesthetized by inhalation of 3.0% isoflurane and positioned supine on a heated platform. After induction, the isoflurane concentration was titrated to 1.0–1.5% to maintain a physiological heart rate of approximately 450 beats per minute and to ensure the absence of pedal reflex. Two-dimensional parasternal long-axis and short-axis views were obtained at the level of the papillary muscles. From the M-mode tracings, left ventricular internal diameter at end-systole (LVIDs) and end-diastole (LVIDd) were measured. Left ventricular end-diastolic volume (LVEDV) and end-systolic volume (LVESV) were calculated. Ejection fraction (EF) was derived using the formula: EF (%) = [(LVEDV – LVESV) / LVEDV] × 100%. Fractional shortening (FS) was calculated as: FS (%) = [(LVIDd – LVIDs) / LVIDd] × 100%. All measurements were performed over at least three consecutive cardiac cycles by an investigator blinded to the experimental groups, and the average value was reported for each parameter.

### 2.5 Cell culture

The human cardiomyocyte lines H9C2 and AC16 were purchased from the National Collection of Authenticated Cell Cultures (Shanghai, China). Both cell lines were cultured in high-glucose Dulbecco's Modified Eagle's Medium (DMEM; Gibco, USA; Cat# C11995500CP) supplemented with 10% (*v*/v) fetal bovine serum (Oricell, New Zealand; Cat# ATPS-10001) and 100 U/mL penicillin/streptomycin (BioSharp, China; Cat# BL302a). Cells were maintained in a humidified incubator at 37 °C with 5% CO₂.

For all in vitro experiments involving chemical treatments, vehicle controls were included. Sodium lactate was dissolved in sterile PBS; control cells received an equal volume of PBS. SAHA was dissolved in DMSO and used at a final DMSO concentration of <0.1% (*v*/v); control cells received an equal volume of DMSO. Cell viability was confirmed to be >90% under all treatment conditions by trypan blue exclusion. For transfection experiments, empty pcDNA3.1 vector and scrambled siRNA served as negative controls for overexpression and knockdown experiments, respectively.

### 2.6 Immunofluorescence analysis

For immunofluorescence staining, cells cultured on coverslips were fixed with 4% paraformaldehyde for 15 min at room temperature, followed by permeabilization with 0.1% Triton X-100 for 10 min. After blocking with 5% bovine serum albumin (BSA) for 1 h, cells were incubated overnight at 4 °C with a primary antibody against CD31. Following three washes with phosphate-buffered saline (PBS), cells were incubated with a corresponding fluorescent dye-conjugated secondary antibody for 1 h at room temperature in the dark. Cell nuclei were counterstained with 4′,6-diamidino-2-phenylindole (DAPI). Images were captured using a fluorescence microscope and analyzed with ImageJ software.

### 2.7 RNA extraction and quantitative Real-Time PCR (qPCR)

Total RNA was extracted from tissues or cultured cells using RNAiso Plus reagent (Takara, Tokyo, Japan) according to the manufacturer's protocol. RNA concentration and purity were assessed spectrophotometrically (NanoDrop, Thermo Fisher Scientific, USA). For reverse transcription, 1 μg of total RNA was converted to complementary DNA (cDNA) using Hifair® III First Strand cDNA Synthesis SuperMix (YEASEN, Shanghai, China).Quantitative real-time PCR (qPCR) was performed on a CFX96 Real-Time PCR Detection System (Bio-Rad, California, USA) using SYBR Green Master Mix (YEASEN). The thermal cycling conditions were as follows: initial denaturation at 95 °C for 5 min, followed by 40 cycles of 95 °C for 10 s and 60 °C for 30 s. A melting curve analysis was performed to verify the specificity of amplification. All primer sequences used in this study are listed in Supplementary Table S1. The relative mRNA expression of target genes was calculated using the 2^(-ΔΔCt) method and normalized to the expression level of β-actin, which served as an internal control.

### 2.8 Plasmid and siRNA transfection

For ACOT1 overexpression, cells were transfected with the pcDNA3.1-ACOT1 plasmid (UniBio, Chongqing, China) using the empty pcDNA3.1 vector as a control. For ACOT1 knockdown, cells were transfected with small interfering RNA targeting ACOT1 (siACOT1), with a scrambled siRNA serving as a negative control. All transfections were performed using jetPRIME transfection reagent (Polyplus, Strasbourg, France) according to the manufacturer's instructions. Briefly, 5 μg of plasmid DNA or 60 nM siRNA was diluted in 500 μL of jetPRIME buffer, followed by the addition of 10 μL of jetPRIME reagent. The mixture was vortexed, briefly centrifuged, and incubated at room temperature for 10 min to allow complex formation. The transfection complexes were then added to cells cultured in fresh medium. After 6 h of incubation at 37 °C under 5% CO₂, the medium was replaced with complete growth medium, and cells were further cultured for 24 h before subsequent experiments.

### 2.9 Western Blot, and Co-immunoprecipitation (Co-IP)

Total protein was extracted from cells or heart tissues using a commercially available protein extraction kit (Kangcheng Bioengineering, Shanghai, China) supplemented with a protease and phosphatase inhibitor cocktail (BioSharp, Hefei, China). Protein concentrations were determined using a bicinchoninic acid (BCA) assay kit (Beyotime, Shanghai, China).

Equal amounts of protein (20–40 μg) were separated by sodium dodecyl sulfate-polyacrylamide gel electrophoresis (SDS-PAGE) and then transferred onto polyvinylidene difluoride (PVDF) membranes (Millipore, Billerica, USA).Membranes were blocked with 5% (*w*/*v*) non-fat milk in TBST for 1 h at room temperature and subsequently incubated overnight at 4 °C with the following primary antibodies: anti-ACOT1 rabbit polyclonal antibody (Cat# 16214–1-AP, Proteintech, Wuhan, China; dilution 1:1000), anti-Pre-SREBP1c rabbit monoclonal antibody (Cat# 9874, Cell Signaling Technology, Danvers, MA, USA; dilution 1:1000), anti-CD36 rabbit monoclonal antibody (Cat# 14347, D8L9T, Cell Signaling Technology; dilution 1:1000), and anti-β-actin rabbit polyclonal antibody (Cat# 20536–1-AP, Proteintech; dilution 1:5000). For lactylation detection, anti-pan-lactyllysine (Pan-Kla) rabbit polyclonal antibody (Cat# STJ111015, St John's Laboratory, London, UK; dilution 1:1000) was used. For acetylation detection, anti-acetylated-lysine (Ac-K-103) mouse monoclonal antibody (Cat# 9681, Cell Signaling Technology; dilution 1:1000) was used. Membranes for Pan-Kla and Ac—K blots were blocked with 5% (*w*/*v*) BSA in TBST; membranes for all other antibodies were blocked with 5% non-fat milk in TBST. After washing, membranes were incubated with appropriate horseradish peroxidase (HRP)-conjugated secondary antibodies for 1 h at room temperature. Protein bands were visualized using an enhanced chemiluminescence (ECL) kit (G2014, ServiceBio, China) and quantified using ImageJ software (National Institutes of Health, USA).

For Co-IP experiments, AC16 cells were co-transfected with the indicated expression plasmids, including Flag-PRE-SREBP1C and its lactylation-deficient mutant FLAG-PRE-SREBP1C-K664/673/716R. At 48 h post-transfection, cells were lysed using IP lysis buffer (P0013, Beyotime) supplemented with protease inhibitors. Lysates were incubated on ice for 10 min and centrifuged at 12,000 ×*g* for 15 min at 4 °C to collect the supernatant. Protein concentration was determined using the BCA method. For each IP reaction, 800 μg of total protein lysate was incubated with 2 μg of the respective primary antibody overnight at 4 °C with gentle rotation. Subsequently, 30 μL of pre-washed Protein A/G agarose beads were added to each sample and incubated for an additional 6 h at 4 °C. A small aliquot (10%) of the initial lysate was saved as the input control. After incubation, beads were collected by centrifugation at 1000 ×*g* for 5 min and washed five times with ice-cold PBS. The immunoprecipitated complexes were eluted by boiling in 2× SDS loading buffer at 100 °C for 5 min and then analyzed by Western blot as described above.

### 2.10 Transcriptomic data analysis

**Fig. 1:**
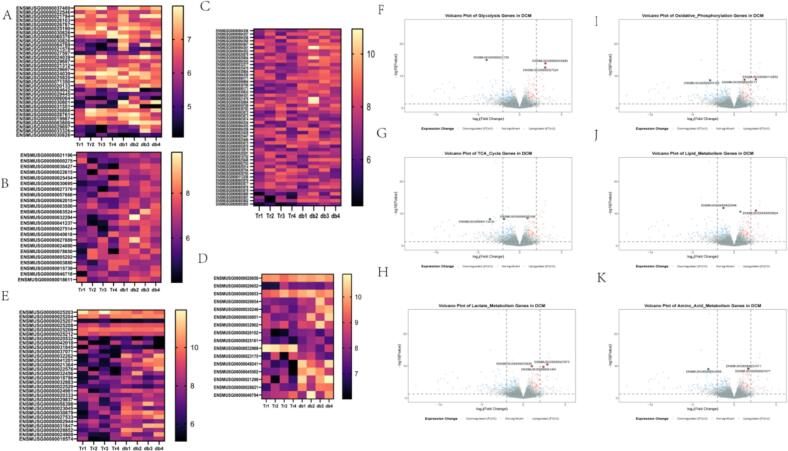
From lipid uptake to oxidation: Comprehensive transcriptomic analysis reveals lipid metabolism as the nexus of metabolic reprogramming in diabetic cardiomyopathy. A. Transcriptomic analysis of amino acid metabolism-related genes in db versus Tr hearts; B. Transcriptomic profiling of glucose metabolism-related genes in db versus Tr myocardium; C-E. Transcriptomic analysis of oxidative phosphorylation-, lactate metabolism-, and lipid metabolism-related genes in diabetic cardiomyopathy; F—H. Transcriptomic profiling of 18 critical genes across six core metabolic pathways in DCM and detailed analysis of lactate metabolism- and TCA cycle-related genes among these pathways; I—K.Analysis of regulatory patterns of the six core metabolic pathways, identification of core driver genes, and schematic illustration of the mechanistic cascade of metabolic reprogramming in DCM.

**Fig. 2:**
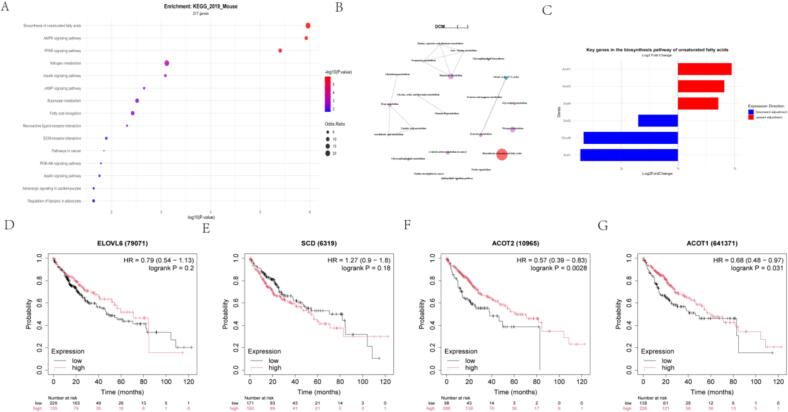
ACOT1 as a critical regulator of lipid metabolism in diabetic cardiomyopathy. A. KEGG pathway enrichment analysis revealing lipid metabolism and energy-sensing signaling pathways as the core transcriptional signature of DCM, with AMPK as a central integrator; B—C. KEGG pathway enrichment analysis of DCM heart transcriptomic data, highlighting biosynthesis of unsaturated fatty acids as the most significantly enriched metabolic pathway and identifying ACOT1 as a critical node; D-G. Prognostic screening of lipid metabolism candidates via KM Plotter, showing elevated ACOT1 expression is associated with improved survival and adequate statistical power throughout the observation period.

Publicly available RNA-sequencing data from cardiac tissue of db/db diabetic mice and control mice were obtained from the Gene Expression Omnibus (GEO) under accession number GSE274500. Differential expression analysis was performed using DESeq2 (v1.38.0) in R (v4.2.0). Genes with |log2 fold change| ≥ 2 and adjusted *P* value <1 × 10^−6^ were considered significantly differentially expressed. KEGG pathway enrichment analysis was performed using clusterProfiler (v4.6.0). This dataset was used exclusively for unbiased screening of dysregulated metabolic pathways and identification of candidate genes (Fig. 1, Fig. 2). All subsequent qPCR validation experiments were performed in independent in vitro and in vivo models as described in the relevant sections below.

### 2.11 Statistical analysis

All quantitative data are presented as mean ± standard error of the mean (SEM), with the biological replicate number indicated by individual animal or sample count (N). Normality and homogeneity of variance were assessed using the Shapiro–Wilk test and Levene's test, respectively. For comparisons between two groups, an unpaired two-tailed Student's *t*-test was used for data meeting assumptions of normality and equal variance; Welch's t-test was applied for normally distributed data with unequal variance; and the non-parametric Mann–Whitney *U* test was employed for data not conforming to a normal distribution. For comparisons among multiple groups, one-way analysis of variance (ANOVA) followed by Tukey's post hoc test was used for normally distributed data with equal variance; the Kruskal–Wallis test with Dunn's post hoc test was applied for non-normally distributed data. *P*-value <0.05 was considered statistically significant. All statistical analyses were performed using GraphPad Prism 8.0 software (GraphPad Software, CA, USA).

## 3 Results

### 3.1 From lipid uptake to oxidation: comprehensive transcriptomic analysis reveals lipid metabolism as the nexus of metabolic reprogramming in diabetic cardiomyopathy

Transcriptomics revealed selective upregulation of tryptophan-kynurenine (TDO2/IDO1) and urea cycle (ASS1/ARG1) in db versus Tr hearts, with stable BCAT2 and glutamate metabolism. However, analysis of 31 amino acid genes showed no global pathological reprogramming in DCM, suggesting these pathways are not central drivers of cardiac metabolic disturbance (Fig. 1a). Transcriptomic profiling revealed significant upregulation of key glycolytic enzymes (PKM, PGAM1, GAPDH) and gluconeogenic regulators (FBP1, FBP2, G6PC) in db compared to Tr myocardium, indicating enhanced glucose metabolic flux through both glycolytic and gluconeogenic pathways. This concurrent activation suggests metabolic reprogramming toward increased glucose turnover in diabetic hearts, potentially reflecting adaptive responses to altered energetic demands or insulin resistance (Fig. 1b). Transcriptomic profiling revealed largely preserved oxidative phosphorylation (OXPHOS) capacity in diabetic cardiomyopathy, with only selective modulation of specific electron transport chain subunits (e.g., mt-Nd5, Sdhb) but no global pathway remodeling, indicating that overall mitochondrial respiratory function remains relatively intact despite diabetic metabolic stress (Fig. 1c). Transcriptomic analysis revealed comprehensive upregulation of lactate metabolism machinery in diabetic cardiomyopathy, highlighted by marked induction of lactate receptors HCAR1, HCAR2, and GPR132, alongside elevated expression of monocarboxylate transporters MCT1 (SLC16A1) and MCT4 (SLC16A3), lactate dehydrogenases LDHB and LDHC, and the MCT chaperone basigin (BSG). These changes indicate enhanced myocardial lactate uptake, oxidation, and signaling in the diabetic heart, with the notable exception of MCT3 (SLC16A8), which exhibited significantly reduced expression, suggesting a selective reprogramming of lactate handling toward cellular uptake rather than efflux in diabetic cardiomyopathy (Fig. 1d). Transcriptomic analysis revealed comprehensive upregulation of lipid metabolism machinery in diabetic cardiomyopathy, characterized by marked induction of fatty acid uptake and transport systems (FABP4, FABP5, SLC27A1/FATP1, SLC27A4/FATP4, ACSL5), augmented fatty acid modification enzymes (SCD1, ELOVL2, ELOVL5, ELOVL6), and supporting lipogenic factors (ACLY, ME1). Additionally, mitochondrial fatty acid oxidation capacity was enhanced through elevated CPT1A and ACADVL expression, indicating a metabolic reprogramming toward increased lipid utilization and trafficking in the diabetic heart (Fig. 1e).

Transcriptomic profiling of 18 critical genes across six core metabolic pathways (glycolysis, lipid metabolism, lactate metabolism, amino acid metabolism, oxidative phosphorylation, and TCA cycle) revealed comprehensive metabolic reprogramming in diabetic cardiomyopathy (DCM), with all pathways exhibiting significant differential expression (|FC| ≥ 2, AdjP <1e-6). Notably, glycolysis and lactate metabolism displayed the most stringent regulatory patterns devoid of low-magnitude changes; specifically, lactate metabolism was uniquely characterized by universal upregulation across all target genes (Log2FC: 1.02–2.87), serving as a direct molecular signature of enhanced anaerobic glycolysis. Conversely, the TCA cycle emerged as the most profoundly suppressed pathway, featuring robust inhibition of ENSMUSG00000113216 (Log2FC = −3.54) alongside coordinated downregulation of complementary genes (Fig. 1f-h).

This reciprocal relationship between TCA cycle suppression and lactate metabolic upregulation suggests a “crippled aerobic-to-anaerobic shift” as the central energetic crisis in DCM. While glycolysis and amino acid metabolism exhibited bidirectional dysregulation (2 upregulated vs. 1 strongly suppressed), and oxidative phosphorylation maintained balanced bidirectional control, lipid metabolism presented heterogeneous modulation with evidence of fine-tuning nodes (|FC| < 2), suggesting nuanced involvement in lipotoxicity rather than bulk pathway remodeling. The identification of ENSMUSG00000021730 (glycolysis, Log2FC = −3.94), ENSMUSG00000113216 (TCA cycle), and ENSMUSG00000027875 (lactate metabolism, Log2FC = 2.87) as core driver genes highlights critical intervention targets. Collectively, these findings delineate a mechanistic cascade wherein TCA cycle failure precipitates compensatory anaerobic metabolism, concurrent substrate utilization disorders, and lipid-induced cytotoxicity, ultimately converging on myocardial energy depletion and DCM progression (Fig. 1i-k).

### 3.2 ACOT1 as a critical regulator of lipid metabolism in diabetic cardiomyopathy

KEGG pathway enrichment analysis reveals that lipid metabolism and energy-sensing signaling pathways constitute the core transcriptional signature of diabetic cardiomyopathy (DCM). The most significantly enriched pathways include biosynthesis of unsaturated fatty acids (SCD1/2/4, ELOVL6, ACOT1/2), PPAR signaling (ADIPOQ, PLIN1, CPT1B), and AMPK signaling (FASN, EIF4EBP1, PCK1), indicating comprehensive reprogramming of cardiac lipid handling. Notably, the co-enrichment of insulin signaling and cAMP-dependent pathways suggests dysregulated hormonal control of myocardial metabolism, wherein AMPK emerges as a central integrator linking energy stress to lipid metabolic adaptation (Fig. 2a).

KEGG pathway enrichment analysis of transcriptomic data from diabetic cardiomyopathy (DCM) hearts revealed that biosynthesis of unsaturated fatty acids represented the most significantly enriched metabolic pathway (*P* = 1.09 × 10^−6^, adjusted *P* = 1.07 × 10^−4^, combined score = 296.67), characterized by coordinated dysregulation of six key genes including stearoyl-CoA desaturases (SCD1/2/4), fatty acid elongase 6 (ELOVL6), and acyl-CoA thioesterases (ACOT1/2). Notably, ACOT1 emerged as a critical node within this lipid-centric signature, also participating in the fatty acid elongation pathway (*P* = 3.74 × 10^−3^) (Fig. 2b-c).

Prognostic screening of the lipid metabolism candidates (Elovl6, Scd, Acot1, and Acot2) via KM Plotter revealed that elevated ACOT1 expression was significantly associated with improved survival (HR = 0.68, 95% CI: 0.48–0.97; log-rank *P* = 0.031). The analysis maintained adequate statistical power throughout the observation period, with patient numbers at risk declining from 138 (low expression) and 226 (high expression) at baseline to 1 in each group at the final follow-up timepoint (Fig. 2d-g).

### 3.3 ACOT1 upregulation in early-stage DCM confers cardioprotection against diabetic cardiomyopathy

Longitudinal profiling of untreated DCM mice revealed a sharp myocardial and serum lactate surge at weeks 18–19, which was followed by progressive LV dilation and fibrosis, while EF remained unchanged (Supplementary Table S1). Based on this lactate surge, we defined weeks 16–18 as the early compensated stage and weeks 19–22 as the decompensated stage.

**Fig. 3:**
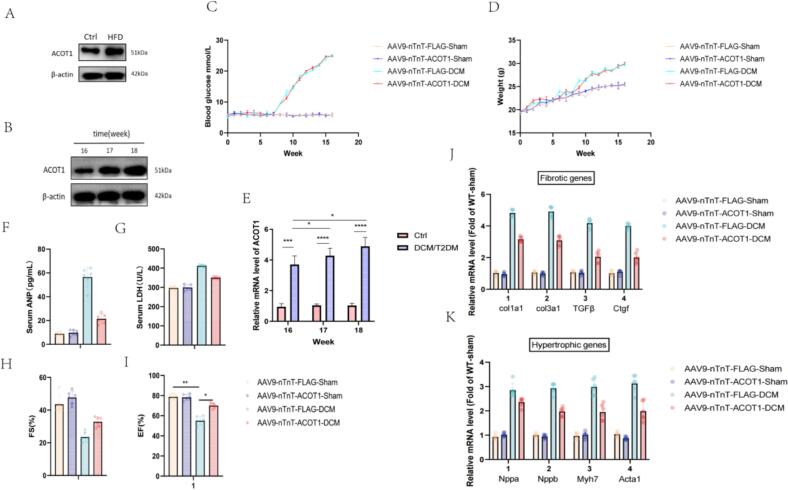
ACOT1 upregulation in early-stage DCM confers cardioprotection. A-C. ACOT1 protein and mRNA expression are progressively induced in DCM hearts from weeks 16–18 (*P* < 0.05–0.01). D-E.HFD/STZ-induced DCM mice exhibit obesity and hyperglycemia. F-G. Cardiac-specific ACOT1 overexpression reduces ANP (*P* < 0.001) and LDH (P < 0.05) in DCM but not control mice. H—I.ACOT1 improves FS and EF in DCM. J-K. ACOT1 attenuates fibrotic (Col1a1, Col3a1, Tgfβ, Ctgf) and hypertrophic (Nppa, Nppb, Myh7, Acta1) gene expression. Data represent mean ± SEM; *P* values determined by Student's *t*-test or one-way ANOVA.

ACOT1 is induced in the early phase of diabetic cardiomyopathy. Western blot analysis demonstrated that cardiac ACOT1 protein expression was significantly elevated in DCM model mice at week 16 compared to control animals (*P* < 0.01, Fig. 3a). Longitudinal profiling across weeks 16–18 revealed a progressive upregulation of ACOT1 protein levels over time (*P* < 0.05, Fig. 3b), paralleled by a corresponding increase in Acot1 mRNA expression (P < 0.05, Fig. 3c), indicating transcriptional activation of this lipid metabolic enzyme during the early compensatory phase of DCM(Data represent mean ± SEM; *n* = 5 mice (independent biological replicates) per time point.).

Establishment of a type 2 diabetes-associated DCM model. High-fat diet (HFD)-fed mice developed obesity and metabolic dysfunction prior to streptozotocin (STZ) administration. Following STZ induction at week 8, DCM mice exhibited sustained hyperglycemia and significantly higher body weights compared to sham-operated controls, confirming successful establishment of a type 2 diabetes-associated DCM phenotype (Fig. 3d-e). Notably, within-group comparisons revealed no significant differences between HFD-fed and standard chow-fed animals prior to STZ treatment, validating the diabetic insult as the primary driver of metabolic and cardiac phenotypic divergence.

ACOT1 overexpression ameliorates cardiac remodeling and preserves contractile function in DCM. To investigate the functional significance of ACOT1 induction, we employed cardiac-specific Acot1 overexpression in DCM mice. This intervention significantly reduced circulating atrial natriuretic peptide (ANP) levels (*P* < 0.001, Fig. 3f) and lactate dehydrogenase (LDH) activity (*P* < 0.05, Fig. 3g), markers of cardiomyocyte stress and injury, respectively. Importantly, these protective effects were DCM-specific, as Acot1 overexpression in non-diabetic control mice did not alter ANP or LDH levels, suggesting that ACOT1-mediated cardioprotection is context-dependent and operative primarily under metabolic stress conditions.

Echocardiographic assessment revealed that Acot1 overexpression significantly improved left ventricular systolic function in DCM mice, as evidenced by increased fractional shortening (FS) and ejection fraction (EF) (Fig. 3h-i). Mechanistically, Acot1 overexpression attenuated cardiac fibrosis, manifesting as downregulation of fibrotic gene markers including Col1a1, Col3a1, Tgfβ, and Ctgf (Fig. 3j). Concurrently, pathological cardiac hypertrophy was mitigated, with reduced expression of hypertrophic genes Nppa, Nppb, Myh7, and Acta1 (Fig. 3k). Collectively, these findings establish that ACOT1 induction during early DCM represents an adaptive compensatory response that constrains adverse cardiac remodeling and preserves contractile function, positioning ACOT1 as a potential therapeutic target for halting DCM progression.

### 3.4 ACOT1 downregulation in late-stage DCM marks transition to decompensated heart failure

**Fig. 4:**
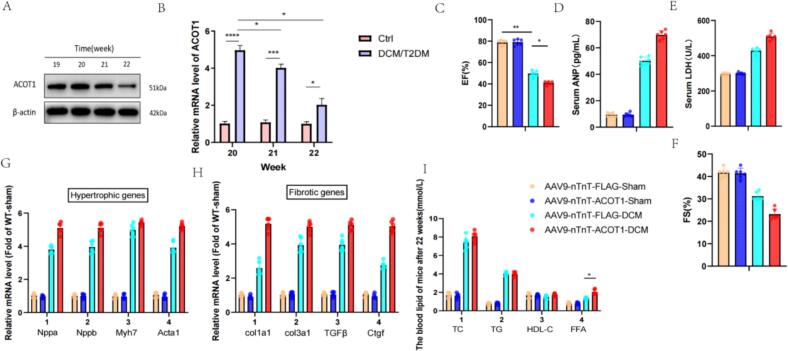
ACOT1 downregulation in late-stage DCM marks transition to decompensated heart failure. A-B. ACOT1 protein and mRNA expression progressively decline in DCM hearts from weeks 19–22 (*P* < 0.05–0.01). C. Sustained ACOT1 overexpression impairs FS and EF in late-stage DCM. D-E. ACOT1 elevation increases ANP and LDH levels (P < 0.05) in DCM but not control mice. F. Schematic illustrating biphasic ACOT1 function. G-H. ACOT1 upregulates fibrotic (Col1a1, Col3a1, Tgfβ, Ctgf) and hypertrophic (Nppa, Nppb, Myh7, Acta1) gene expression. I.ACOT1 selectively elevates circulating FFA (P < 0.05) without altering TC, TG, or HDL—C. Data represent mean ± SEM; *P* values determined by one-way ANOVA or Student's *t*-test.

ACOT1 expression declines during DCM progression to advanced stages. Western blot analysis revealed progressive downregulation of cardiac ACOT1 protein expression from weeks 19–22 in DCM mice (P < 0.05–0.01, Fig. 4a), with corresponding reductions in Acot1 mRNA levels at weeks 20–22 (*P* < 0.05, Fig. 4b). This temporal decline contrasts sharply with the early compensatory upregulation observed at weeks 16–18 (Fig. 3), identifying late-stage ACOT1 loss as a molecular hallmark of DCM decompensation.

ACOT1 overexpression spanning both early and decompensated stages exacerbates cardiac dysfunction. To determine whether ACOT1 expression that fails to undergo its natural decline influences disease trajectory, we employed cardiac-specific Acot1 overexpression initiated at week 16 and maintained through week 22. Paradoxically, sustained ACOT1 elevation significantly impaired left ventricular systolic function, as evidenced by decreased fractional shortening (FS) and ejection fraction (EF) (Fig. 4c). This functional deterioration was accompanied by exacerbated cardiomyocyte injury, manifesting as increased circulating ANP (*P* < 0.05, Fig. 4d) and LDH (P < 0.05, Fig. 4e) levels. Importantly, these deleterious effects were DCM-specific, as Acot1 overexpression in non-diabetic control mice did not alter cardiac function or injury markers, indicating that the pathological consequences of sustained ACOT1 activity are contingent upon the diabetic metabolic milieu.

ACOT1 persistence Modulates adverse cardiac remodeling and lipotoxicity. Mechanistically, sustained Acot1 overexpression in late-stage DCM promoted pathological cardiac remodeling, evidenced by upregulation of fibrotic gene markers (Col1a1, Col3a1, Tgfβ, Ctgf, Fig. 4g) and hypertrophic gene signatures (Nppa, Nppb, Myh7, Acta1, Fig. 4h). Metabolic profiling at week 22 revealed that Acot1 overexpression specifically elevated circulating free fatty acid (FFA) levels (*P* < 0.05, Fig. 4i), without significantly altering total cholesterol (TC), triglycerides (TG), or high-density lipoprotein cholesterol (HDL—C). This selective FFA accumulation suggests that persistent ACOT1 activity in advanced DCM disrupts lipid homeostasis, promoting lipotoxicity-driven cardiac dysfunction. Collectively, these findings establish ACOT1 as a biphasic modulator of DCM progression—cardioprotective when transiently induced during early metabolic stress, but maladaptive when its expression persists rather than undergoing the natural decline that accompanies the transition to decompensation.

### 3.5 ACOT1 sensitizes SREBP-1c activation to lactate stimulation in cardiomyocytes

**Fig. 5:**
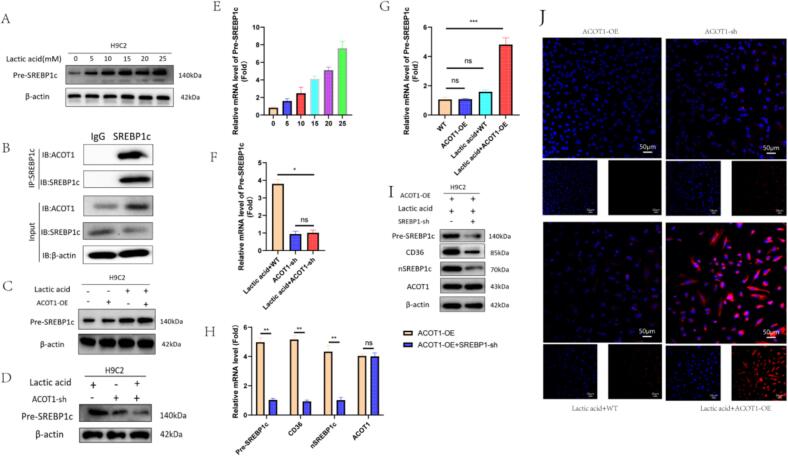
ACOT1 modulates lactate-induced SREBP-1c activation and CD36 upregulation. A. Lactate dose-dependently induces Pre-SREBP1c in H9C2 cells. B. Co-immunoprecipitation showing physical interaction between endogenous ACOT1 and SREBP-1c in AC16 cells under high-lactate conditions. C. ACOT1 overexpression synergizes with lactate to enhance Pre-SREBP1c protein levels. D. ACOT1 knockdown attenuates lactate-induced Pre-SREBP1c expression. *E*-G. Lactate elevates Srebp1c mRNA, with ACOT1 overexpression producing synergistic activation (~2.5-fold). H. SREBP-1c knockdown reverses ACOT1-mediated induction of Srebp1c, Cd36, and nSrebp1c. I. Combined ACOT1 overexpression and lactate maximally induces Pre-SREBP1c, nSREBP1c, and CD36 proteins. I. Immunofluorescence confirms ACOT1 is required for lactate-driven CD36 upregulation. Data represent mean ± SEM; *P* values determined by one-way ANOVA.

Lactate induces SREBP-1c precursor expression in a concentration-dependent manner. In H9C2 cardiomyocytes, exogenous lactate treatment dose-dependently upregulated precursor SREBP-1c (Pre-SREBP1c) protein levels, establishing lactate as a direct metabolic cue activating lipogenic transcriptional programs (Fig. 5a).To investigate whether ACOT1 physically interacts with SREBP-1c, we performed co-immunoprecipitation in H9C2 cardiomyocytes under high-lactate conditions. Endogenous ACOT1 was specifically detected in SREBP-1c immunoprecipitates but not in control IgG precipitates (Fig. 5b), demonstrating a physical interaction between these two proteins in cardiomyocytes. This interaction provides a mechanistic basis for ACOT1's ability to facilitate SREBP-1c activation in response to lactate.

ACOT1 synergizes with lactate to amplify SREBP-1c activation. Under lactate-free conditions, ACOT1 overexpression alone had minimal effect on Pre-SREBP1c expression. However, in the presence of lactate, ACOT1 overexpression markedly potentiated Pre-SREBP1c protein levels, with the combination treatment producing the strongest induction (Fig. 5c). Conversely, ACOT1 knockdown significantly attenuated lactate-mediated Pre-SREBP1c upregulation, identifying ACOT1 as an essential molecular mediator linking lactate signaling to SREBP-1c activation (Fig. 5d).

Transcriptional validation of ACOT1-lactate-SREBP-1c axis. Quantitative PCR confirmed that lactate elevated Srebp1c mRNA levels (Fig. 5e), and this induction was abrogated by ACOT1 silencing (Fig. 5f), paralleling protein-level observations. Notably, while lactate alone modestly increased Srebp1c transcription, combined ACOT1 overexpression and lactate treatment produced synergistic activation (~2.5-fold enhancement), whereas ACOT1 overexpression alone was ineffective (Fig. 5g). These findings demonstrate that ACOT1 functions as a lactate-dependent co-activator of SREBP-1c transcription.

SREBP-1c mediates ACOT1-induced lipogenic gene expression. Epistasis analysis revealed that SREBP-1c knockdown reversed ACOT1-mediated induction of Srebp1c, Cd36, and mature nuclear SREBP-1c (nSrebp1c) without affecting Acot1 expression itself, positioning SREBP-1c downstream of ACOT1 (Fig. 5h). Western blot analysis confirmed that maximal Pre-SREBP1c, nSREBP1c, and CD36 protein induction occurred only under combined ACOT1 overexpression and lactate stimulation, with CD36 specifically requiring this dual input (Fig. 5i).

ACOT1 is indispensable for lactate-driven CD36 membrane localization. Immunofluorescence staining demonstrated that lactate alone modestly induced CD36 expression. ACOT1 overexpression markedly potentiated this effect, whereas ACOT1 knockdown virtually abolished lactate-induced CD36 upregulation (Fig. 5j). Collectively, these data establish an ACOT1-SREBP-1c-CD36 regulatory axis wherein ACOT1 acts as a metabolic sensor that modulateSREBP-1c activation and downstream fatty acid uptake machinery in response to lactate accumulation, providing a mechanistic basis for the lipotoxic phenotype observed in sustained ACOT1 expression during late-stage DCM.

### 3.6 SREBP-1c undergoes lactylation at conserved lysine 716 residue

**Fig. 6:**
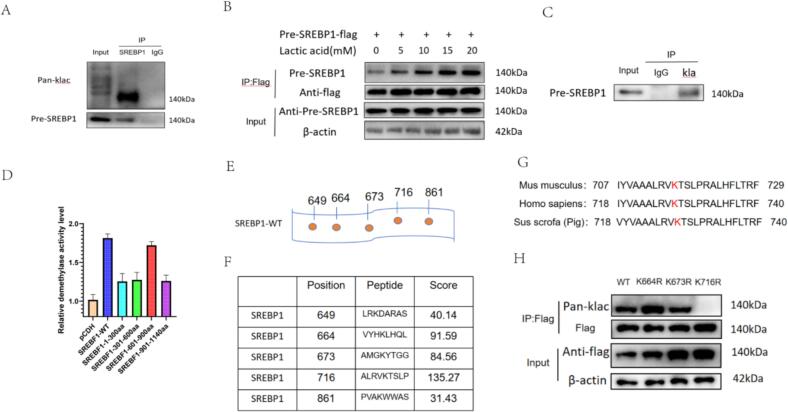
SREBP-1c undergoes lactylation at conserved lysine 716 residue. A-C. Immunoprecipitation and Pan-Kla detection experiments confirming Pre-SREBP1c is post-translationally modified by lactylation, with lactylation intensity increasing concentration-dependently with exogenous lactate and signal specificity verified by IgG controls; E-H. Computational prediction of candidate lactylation sites on Pre-SREBP1c, mass spectrometry analysis identifying K716 as the predominant lactylation site, evolutionary conservation analysis of K716 across species, and site-directed mutagenesis confirming K716 as the primary lactylation residue.

Pre-SREBP1c is post-translationally modified by lactylation. Immunoprecipitation of Pre-SREBP1c-Flag followed by pan-lactyllysine antibody (Pan-Kla) detection revealed robust lactylation signal on Pre-SREBP1c, establishing this lipogenic transcription factor as a target of lactate-derived metabolic regulation (Fig. 6a). The lactylation intensity increased concentration-dependently with exogenous lactate (0–20 mM), demonstrating direct responsiveness to cellular lactate accumulation (Fig. 6b). Specificity was confirmed by selective detection of Pan-Kla signal in Flag-IP samples but not IgG controls (Fig. 6c).

K716 is the predominant lactylation site on Pre-SREBP1c. Computational prediction identified five candidate lysine residues within the 601–900 amino acid region (K649, K664, K673, K716, K861) as potential lactylation sites (Fig. 6e). Mass spectrometry analysis revealed K716 as the highest-scoring modification site (score: 135.27, Fig. 6f), with evolutionary conservation across human, mouse, and pig orthologs underscoring its functional significance (Fig. 6g). Site-directed mutagenesis demonstrated that K716R substitution markedly attenuated Pan-Kla signal intensity compared to K664R or K673R mutations, establishing K716 as the primary lactylation residue mediating SREBP-1c metabolic regulation (Fig. 6h). These findings identify SREBP-1c K716 lactylation as a conserved post-translational mechanism linking lactate accumulation to lipogenic transcriptional activation in diabetic cardiomyopathy.

To validate the specificity of the lactylation signal detected on Pre-SREBP1c, we performed two independent control experiments. First, pre-incubation of the Pan-Kla antibody with excess lactylated BSA completely abolished the lactylation signal in Flag-Pre-SREBP1c immunoprecipitates (Supplementary Fig. 1), confirming that the antibody specifically recognizes lactylated lysine residues. Second, to exclude potential cross-reactivity with acetylation—a modification structurally similar to lactylation—we treated Flag-Pre-SREBP1c-expressing H9C2 cells with lactate (20 mM) in the presence or absence of the HDAC inhibitor SAHA (1 μM). SAHA treatment markedly increased the pan-acetyl-lysine (Ac—K) signal but did not enhance the Pan-Kla signal beyond the level induced by lactate alone (Supplementary Fig. 2). This indicates that the Pan-Kla signal reflects bona fide lactylation rather than acetylation.

### 3.7 ACOT1 modulates lipotoxicity through SREBP-1c K716 lactylation-dependent CD36 upregulation

**Fig. 7:**
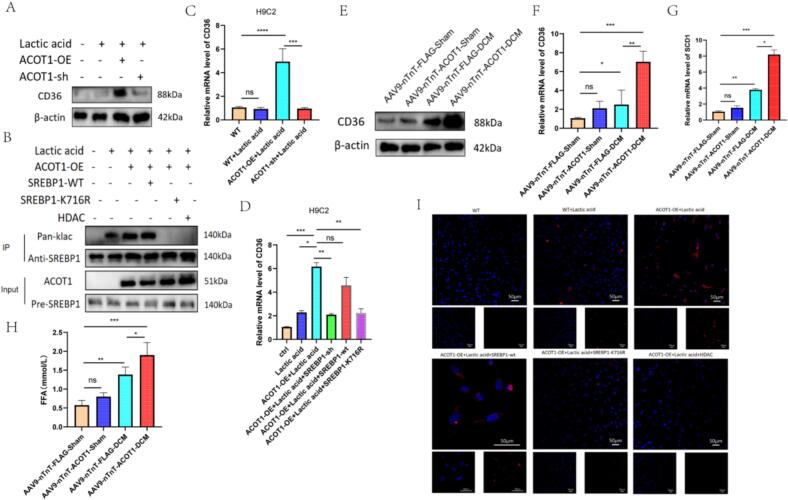
ACOT1 Modulates lipotoxicity via SREBP-1c K716 lactylation-dependent CD36 upregulation. A. ACOT1 enhances Pre-SREBP1c lactylation and CD36 expression; knockdown abrogates this effect. B.K716R mutation or HDAC treatment blocks ACOT1-SREBP-1c-CD36 axis. C.ACOT1 synergizes with lactate to induce CD36 mRNA; knockdown eliminates lactate responsiveness.D.ACOT1-lactate cooperation requires intact SREBP-1c and K716. *E*-F.ACOT1 upregulates cardiac CD36 protein and mRNA specifically in DCM. G.ACOT1 coordinately induces CD36 (uptake) and SCD1 (synthesis). H. Myocardial FFA peaks with sustained ACOT1 in late DCM. I. Immunofluorescence confirms K716 lactylation is essential for CD36 membrane localization. Data represent mean ± SEM; *P* values determined by one-way ANOVA.

ACOT1 promotes SREBP-1c lactylation to activate lipogenic transcription. ACOT1 overexpression enhanced Pre-SREBP1c lactylation, particularly at K716, thereby amplifying its transcriptional activity and upregulating the downstream fatty acid transporter CD36; conversely, ACOT1 knockdown abrogated this regulatory axis (Fig. 7a). The functional necessity of K716 lactylation was validated by site-specific mutagenesis: K716R substitution blocked ACOT1-mediated SREBP-1c activation and CD36 induction, while HDAC-mediated delactylation similarly suppressed CD36 expression, establishing lysine lactylation as an obligate modification for SREBP-1c-driven lipogenesis (Fig. 7b).

Lactate-dependent, ACOT1-gated CD36 transcription requires intact SREBP-1c K716. In H9C2 cardiomyocytes, lactate alone exerted minimal effect on CD36 mRNA expression. However, ACOT1 overexpression synergized with lactate to markedly induce CD36 transcription, whereas ACOT1 silencing completely abolished lactate responsiveness (Fig. 7c). This cooperative effect was strictly dependent on SREBP-1c expression and K716 lactylation status, as both SREBP-1c knockdown and K716R mutation eliminated ACOT1-mediated CD36 upregulation (Fig. 7d).

ACOT1-SREBP-1c-CD36 axis operates in a pathological context-dependent manner. In vivo studies demonstrated that ACOT1 overexpression significantly enhanced cardiac CD36 protein and mRNA expression specifically under DCM conditions, with minimal impact in healthy hearts (Fig. 7e–7f). This pathological selectivity was accompanied by coordinate upregulation of SCD1, indicating that ACOT1 simultaneously promotes fatty acid uptake (via CD36) and de novo lipogenesis (via SREBP-1c/SCD1), thereby establishing a feed-forward lipotoxic loop (Fig. 7g). Consistent with this mechanism, myocardial FFA content peaked in late-stage DCM mice with sustained ACOT1 expression, significantly exceeding all control groups (Fig. 7h).

To directly assess whether ACOT1-driven CD36 upregulation leads to accumulation of lipotoxic lipid intermediates, we measured intracellular diacylglycerol (DAG) content by ELISA in H9C2 cardiomyocytes. Lactate treatment alone did not significantly increase DAG levels compared with untreated WT cells (1.115 ± 0.045 vs. 1.054 ± 0.049 pmol/μg protein, *P* > 0.05). However, ACOT1 overexpression combined with lactate treatment markedly increased DAG content to approximately 3.3-fold of WT levels (3.309 ± 0.116 pmol/μg protein, *P* < 0.001 vs. WT + Lactic acid). Importantly, ACOT1 knockdown in the presence of lactate reduced DAG levels to near-baseline (1.497 ± 0.105 pmol/μg protein, P < 0.001 vs. ACOT1-OE + Lactic acid; P > 0.05 vs. WT + Lactic acid) (Supplementary Fig. S4). These data demonstrate that ACOT1 overexpression, in a lactate-rich environment, drives intracellular accumulation of the lipotoxic intermediate DAG, providing direct biochemical evidence linking the ACOT1-SREBP-1c-CD36 axis to lipotoxic stress.

K716 lactylation is indispensable for ACOT1-SREBP-1c-CD36 signaling. Immunofluorescence visualization confirmed negligible CD36 signal in control H9C2 cells and minimal induction by lactate alone. ACOT1 overexpression combined with lactate produced robust CD36 membrane localization, which was preserved by wild-type SREBP-1c reconstitution but completely abolished by K716R mutant introduction. HDAC-mediated delactylation similarly extinguished CD36 signal, corroborating that K716 lactylation constitutes the molecular switch governing ACOT1-dependent lipid uptake in diabetic cardiomyopathy (Fig. 7i).

## 4 Discussion

Our comprehensive transcriptomic analysis of diabetic cardiomyopathy (DCM) reveals several interconnected mechanisms underlying myocardial metabolic reprogramming, shedding new light on the pathogenesis of this complex cardiovascular complication and identifying promising therapeutic targets [30]. These findings not only expand our understanding of the molecular basis of DCM-related metabolic disturbance but also provide compelling evidence for the stage-specific regulation of cardiac function by lipid metabolic regulators, with ACOT1 emerging as a key nodal molecule. Contrary to the prevailing paradigm that global mitochondrial respiratory collapse Modulates DCM, our data demonstrate preserved overall oxidative phosphorylation (OXPHOS) capacity, with only selective modulation of specific electron transport chain subunits, highlighting that the central energetic crisis in DCM stems from a “crippled aerobic-to-anaerobic shift” rather than widespread mitochondrial dysfunction [27], [30], [31]. This shift is characterized by profound TCA cycle suppression and universal upregulation of lactate metabolic machinery, establishing lactate as an active signaling molecule in DCM—a finding consistent with the growing recognition of lactate as a key metabolic cue in cardiac disease [32], [33]. Concurrent upregulation of glycolytic and gluconeogenic regulators further indicates increased glucose turnover, likely an adaptive response to impaired aerobic metabolism and insulin resistance, while amino acid metabolism shows only selective dysregulation of tryptophan-kynurenine and urea cycles without global remodeling, excluding it as a central driver and refining prior hypotheses implicating broader amino acid disturbance in DCM [34], [35].

Notably, lipid metabolism serves as the nexus of DCM metabolic reprogramming, displaying heterogeneous modulation consistent with a nuanced role in lipotoxicity—marked by upregulated fatty acid uptake, modification, and oxidation [36]. KEGG pathway analysis identifies biosynthesis of unsaturated fatty acids as the most significantly dysregulated pathway, with ACOT1 as a critical regulatory node, which aligns with accumulating evidence that dysregulated fatty acid handling Modulates cardiomyocyte injury in DCM. Our longitudinal and functional studies further reveal a context-dependent biphasic role of ACOT1 in DCM progression: transient upregulation in early DCMexerts cardioprotective effects, reducing cardiomyocyte injury, attenuating cardiac fibrosis and hypertrophy, and preserving left ventricular systolic function, with no impact on healthy hearts. Clinically, KM Plotter data link elevated ACOT1 expression to improved DCM patient survival (HR = 0.68, 95% CI: 0.48–0.97; log-rank *P* = 0.031), providing translational relevance to this early adaptive response. Conversely, ACOT1 expression declines in late DCM, and overexpression that fails to undergo this natural decline exacerbates cardiac dysfunction, promotes adverse remodeling, and Modulates selective free fatty acid (FFA) accumulation—mirroring the biphasic regulatory patterns of other metabolic mediators in cardiac disease and highlighting the temporal complexity of adaptive versus maladaptive metabolic responses in DCM [37].

A key question raised by our study is how ACOT1—canonically a thioesterase—facilitates SREBP-1c K716 lactylation. Our co-immunoprecipitation data demonstrate that ACOT1 physically interacts with SREBP-1c in cardiomyocytes under high-lactate conditions, providing a mechanistic basis for this functional association. While ACOT1 is not itself a lactyltransferase, its physical association with SREBP-1c suggests it may function as a scaffolding protein that facilitates enzyme-substrate proximity, or may modulate the local metabolic microenvironment to favor lactylation. Future structural and biochemical studies are warranted to define the precise molecular architecture of this interaction and its enzymatic consequences.

A limitation of our in vivo gain-of-function studies warrants explicit discussion. In our AAV9-mediated overexpression experiments, ACOT1 overexpression was initiated at week 16 (early compensated stage) and maintained through week 22 (decompensated stage). This design indicates the consequences of ACOT1 expression that fails to undergo the natural decline documented in Supplementary Table S1, but cannot formally distinguish whether the observed adverse effects result from (a) ACOT1 activity extending beyond its physiological window, or (b) high-level ACOT1 expression per se regardless of disease stage. Definitive resolution would require an experiment in which ACOT1 overexpression is initiated specifically at week 19—after endogenous ACOT1 has naturally declined—using delayed AAV delivery or a cardiac-specific inducible expression system (e.g., Tet-On). We have therefore constrained our conclusions to reflect that failure of ACOT1's natural decline, rather than overexpression alone, is the key pathological event.

The biphasic behavior of ACOT1—protective during early compensation, deleterious when expression persists into decompensation—parallels the stage-dependent roles documented for several other metabolic regulators in the failing heart. PPARα, a master regulator of fatty acid oxidation, is cardioprotective when activated early in diabetic hearts but exacerbates lipotoxicity when chronically overexpressed, a pattern attributed to the temporal shift from adaptive substrate utilization to maladaptive lipid overload. Similarly, SIRT1 exerts cardioprotection via deacetylation of metabolic targets during early metabolic stress, yet sustained SIRT1 activation can paradoxically impair cardiac function through excessive suppression of glycolytic and mitochondrial gene programs. The biphasic behavior of ACOT1 adds a new dimension to this paradigm: ACOT1's functional switch is gated not merely by its expression level, but by the metabolic milieu—specifically, the lactate surge that accompanies the transition to decompensation. This lactate-dependent, lactylation-mediated mechanism distinguishes ACOT1 from PPARα and SIRT1, whose biphasic effects are primarily driven by transcriptional or post-translational feedback loops independent of glycolytic metabolite accumulation. Whether a unifying principle underlies these functionally analogous but mechanistically distinct biphasic regulators remains an intriguing question for future investigation.

The most significant contribution of this study lies in elucidating the mechanistic basis of ACOT1's biphasic role through the identification of a novel lactate-dependent ACOT1-SREBP-1c-CD36 regulatory axis, gated by lysine lactylation of SREBP-1c—a post-translational modification that links metabolic status to transcriptional regulation [38]. CD36, a transmembrane glycoprotein belonging to the class B scavenger receptor family, functions as a key mediator of fatty acid uptake and transport in cardiomyocytes, playing a pivotal role in regulating cellular lipid homeostasis by facilitating the internalization of long-chain free fatty acids (FFAs) into cells [39], [40], [41]. Lactate dose-dependently upregulates precursor SREBP-1c (Pre-SREBP1c), and ACOT1 acts as a lactate-dependent co-activator, synergizing with lactate to amplify SREBP-1c activation while knockdown abrogates this effect. Mass spectrometry identifies the conserved lysine 716 (K716) as the predominant lactylation site on SREBP-1c, and site-directed mutagenesis confirms that K716 lactylation is indispensable for SREBP-1c activation and downstream CD36 upregulation. ACOT1 promotes K716 lactylation to enhance CD36-mediated FFA uptake, operating specifically under DCM conditions: in early DCM, this axis fine-tunes lipid utilization to compensate for impaired aerobic metabolism, while persistent activation in late DCM creates a feed-forward lipotoxic loop that overwhelms cardiomyocytes [42], [43]. This mechanism suggests a potential link between the ‘crippled aerobic-to-anaerobic shift’ and lipotoxicity, providing new insights into the molecular crosstalk between lactate and lipid metabolism in DCM. Definitive causal evidence at the whole-animal level will require future studies employing metabolic flux analyses and cardiac-specific inducible genetic models [44], [45].

New evidence indicates that mitochondrial quality control—encompassing the UPR, mitophagy, and dynamics—serves as a convergent pathway through which metabolic stressors cause cardiomyocyte injury [46], [47], [48]. Recent studies have demonstrated that axes such as NDUFS4-SIRT5-DUSP1, PIEZO1-TMBIM6-PHB2, and Piezo1-VDAC1 govern mitochondrial homeostasis in various cardiac injury models by modulating mtUPR, energy metabolism, and organelle crosstalk [49], [50], [51], [52]. In parallel, the ACOT1-SREBP-1c-CD36 axis identified here may drive mitochondrial dysfunction through excessive FFA uptake and DAG accumulation, overwhelming the ETC and promoting incomplete β-oxidation [53], [54]. Whether SREBP-1c K716 lactylation directly intersects with mitochondrial quality control sensors such as SIRT5, PGC-1α, or ATF5 warrants future investigation [55], [56].

These findings have important translational implications for DCM therapy [30]. First, they identify ACOT1 and SREBP-1c K716 lactylation as stage-specific therapeutic targets: boosting ACOT1 activity in early DCM may halt adverse cardiac remodeling, while inhibiting ACOT1 or SREBP-1c K716 lactylation in late DCM could disrupt the lipotoxic loop and prevent decompensated heart failure. Given the conservation of SREBP-1c K716 across species, this lactylation site represents a phylogenetically conserved target with potential relevance to human DCM. Second, our study refines the therapeutic paradigm for DCM, emphasizing the importance of stage-specific metabolic intervention rather than universal targeting of lipid or glucose metabolism. Despite these advances, several limitations and future directions warrant consideration. While our experiments demonstrate the validity of these mechanisms in a mouse model of type 2 DCM, clinical validation in human cardiac tissues and alternative DCM models (e.g., type 1 diabetes) is essential to confirm generalizability. Additionally, the upstream signals driving ACOT1's initial upregulation and subsequent downregulation during DCM progression remain unclear, and further studies are needed to explore other potential lactylated substrates of ACOT1 beyond SREBP-1c. Future research should also investigate potential synergistic interactions between ACOT1-targeted therapy and existing DCM interventions to optimize therapeutic efficacy.

## 5 Conclusion

In conclusion, our transcriptomic and functional analyses demonstrate that a “crippled aerobic-to-anaerobic shift” is the central energetic crisis in diabetic cardiomyopathy (DCM), with lipid metabolism serving as the nexus of metabolic reprogramming. ACOT1 acts as a context-dependent biphasic regulator in DCM progression: transient upregulation in early-stage DCM exerts cardioprotection, while failure of its natural decline during decompensation is associated with lipotoxicity and cardiac dysfunction.ACOT1 mediates DCM progression via a novel lactate-dependent regulatory axis: physical interaction between ACOT1 and SREBP-1c facilitates the latter's lactylation at the conserved K716 residue, upregulating CD36 and driving fatty acid accumulation. Our findings identify ACOT1 and SREBP-1c K716 lactylation as potential stage-specific therapeutic targets for DCM, providing a concise mechanistic framework for precision metabolic intervention in this disease.

## CRediT authorship contribution statement

**Zeyu Liao:** Writing – review & editing, Writing – original draft, Methodology, Formal analysis, Data curation, Conceptualization. **Yahui Fu:** Writing – review & editing, Writing – original draft, Supervision, Software. **Ran Li:** Visualization, Validation, Funding acquisition.

## Ethical statement

All animal experiments were performed in accordance with the institutional guidelines and approved by the Experimental Animal Ethics Committee of Zhengzhou University Medical School (Approval No.ZZU-LAEC-2025-458). All procedures involving mice were conducted in compliance with the ARRIVE guidelines and the National Institutes of Health Guide for the Care and Use of Laboratory Animals. Every effort was made to minimize animal suffering and reduce the number of animals used.

The transcriptomic data reanalyzed in this study were obtained from a publicly available dataset (GEO accession number: GSE274500). No additional ethical approval was required for the use of these de-identified, publicly accessible data.

## Funding statement

This work was supported by the 10.13039/501100001809National Natural Science Foundation of China (Grant No. 82100252). The funding body had no role in the design of the study, data collection, analysis, interpretation, or writing of the manuscript.

## Declaration of competing interest

The authors declare that there are no conflicts of interest regarding the publication of this manuscript. No financial, editorial, or personal relationships exist that could inappropriately influence the work presented here, including but not limited to consultancy, stock ownership, equity interests, patent or licensing arrangements, or any other form of competing interest.

## Appendix A Supplementary data

Supplementary Fig. 1 Lactylation blocking peptide competition assay confirms Pan-Kla antibody specificity.

Supplementary Fig. 2 HDAC inhibitor plus lactate control excludes acetylation cross-reactivity of the Pan-Kla antibody.

Supplementary Fig. S3 Western blot images with IgG controls for all Co-IP experiments.

Supplementary Fig. S4 ACOT1 overexpression increases intracellular diacylglycerol (DAG) accumulation.

Supplementary Table S1 Longitudinal metabolic, functional, and histopathological parameters in HFD/STZ-induced DCM mice during natural disease progression.(*n* = 5)

Supplementary Table S2 Primer sequences used for qRT-PCR.
