# Supplementary material for: ACOT1-specific expression modulates metabolic reprogramming in diabetic cardiomyopathy: The role of SREBP1c lactylation in CD36-mediated lipotoxicity
Source: Am Heart J Plus. 2026 Jun 6;67:100809. doi: 10.1016/j.ahjo.2026.100809 (PMC13260214; doi:10.1016/j.ahjo.2026.100809)

Supplementary Figure 1. Lactylation blocking peptide competition assay confirms Pan-Kla antibody specificity.

Flag-Pre-SREBP1c was immunoprecipitated from H9C2 cells treated with lactate (20 mM, 24 h). Immunoprecipitates were resolved by SDS-PAGE and immunoblotted with Pan-Kla antibody with or without pre-incubation with excess lactylated BSA. The lactylation signal was completely abolished in the blocked lane, confirming that the Pan-Kla antibody specifically detects lactylated lysine residues. IB, immunoblotting; IP, immunoprecipitation.


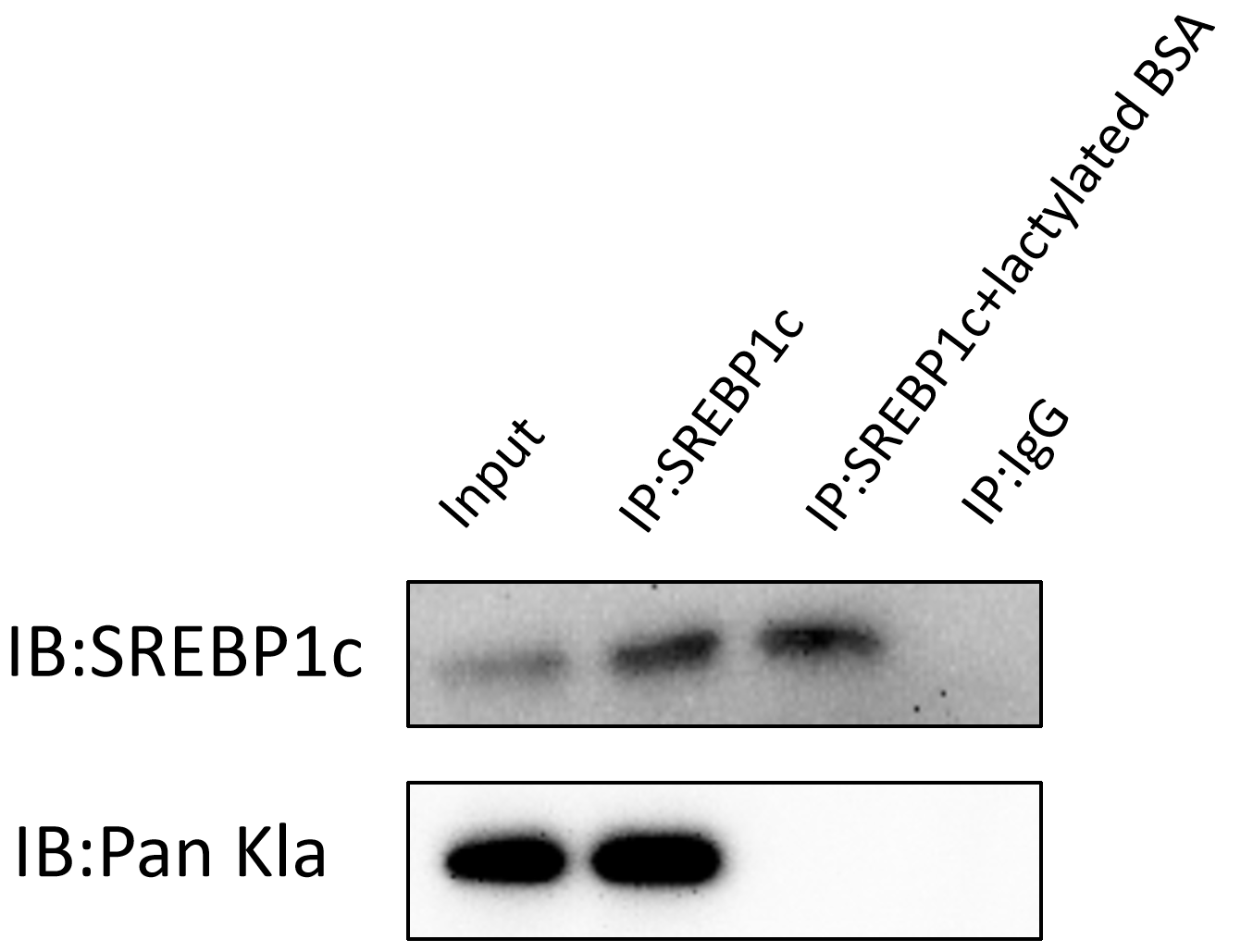

Supplement: Supplementary Fig. 1 — Lactylation blocking peptide competition assay confirms Pan-Kla antibody specificity. [file mmc1.docx]
