# Supplementary material for: ACOT1-specific expression modulates metabolic reprogramming in diabetic cardiomyopathy: The role of SREBP1c lactylation in CD36-mediated lipotoxicity
Source: Am Heart J Plus. 2026 Jun 6;67:100809. doi: 10.1016/j.ahjo.2026.100809 (PMC13260214; doi:10.1016/j.ahjo.2026.100809)

Supplementary Figure 2. HDAC inhibitor plus lactate control excludes acetylation cross-reactivity of the Pan-Kla antibody.

H9C2 cells expressing Flag-Pre-SREBP1c were treated with lactate (20 mM) alone or in combination with the HDAC inhibitor SAHA (1 µM) for 24 h. Flag immunoprecipitates were immunoblotted in parallel with Pan-Kla and pan-acetyl-lysine (Ac-K) antibodies. SAHA markedly increased the Ac-K signal but did not further enhance the Pan-Kla signal beyond that induced by lactate alone, confirming that the Pan-Kla antibody specifically detects lactylation rather than acetylation.


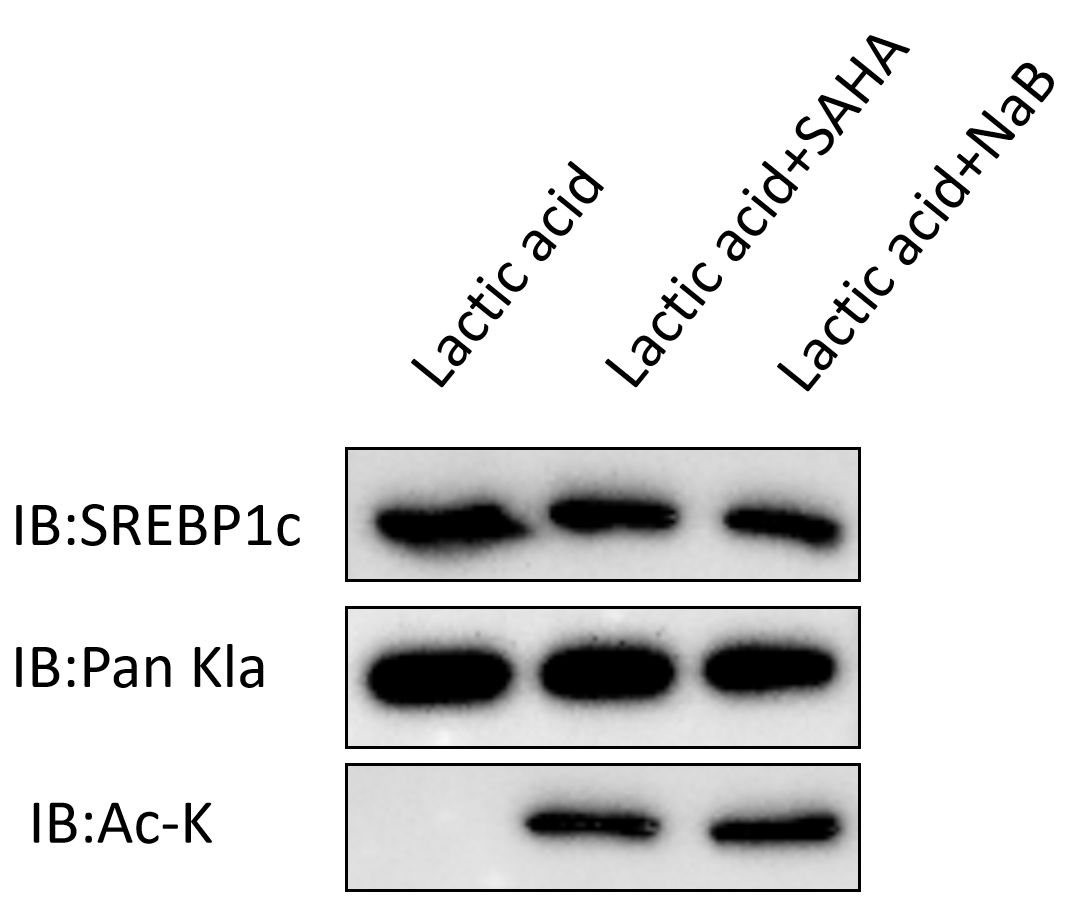

Supplement: Supplementary Fig. 2 — HDAC inhibitor plus lactate control excludes acetylation cross-reactivity of the Pan-Kla antibody. [file mmc2.docx]
