# Supplementary figures and images for: ACOT1-specific expression modulates metabolic reprogramming in diabetic cardiomyopathy: The role of SREBP1c lactylation in CD36-mediated lipotoxicity
Source: Am Heart J Plus. 2026 Jun 6;67:100809. doi: 10.1016/j.ahjo.2026.100809 (PMC13260214; doi:10.1016/j.ahjo.2026.100809)

Supplementary Figure S3.

Western blot images with IgG controls for all Co-IP experiments.


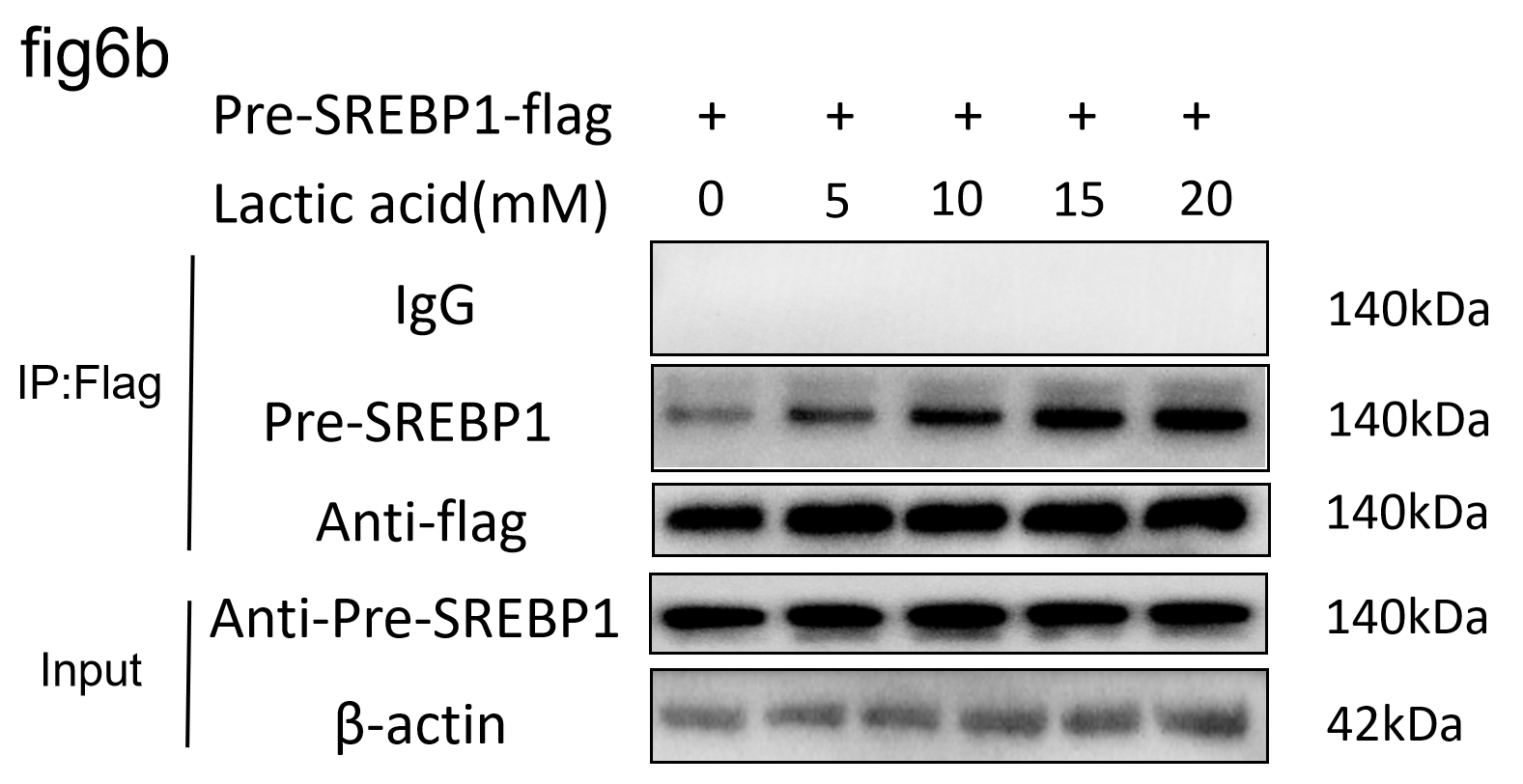

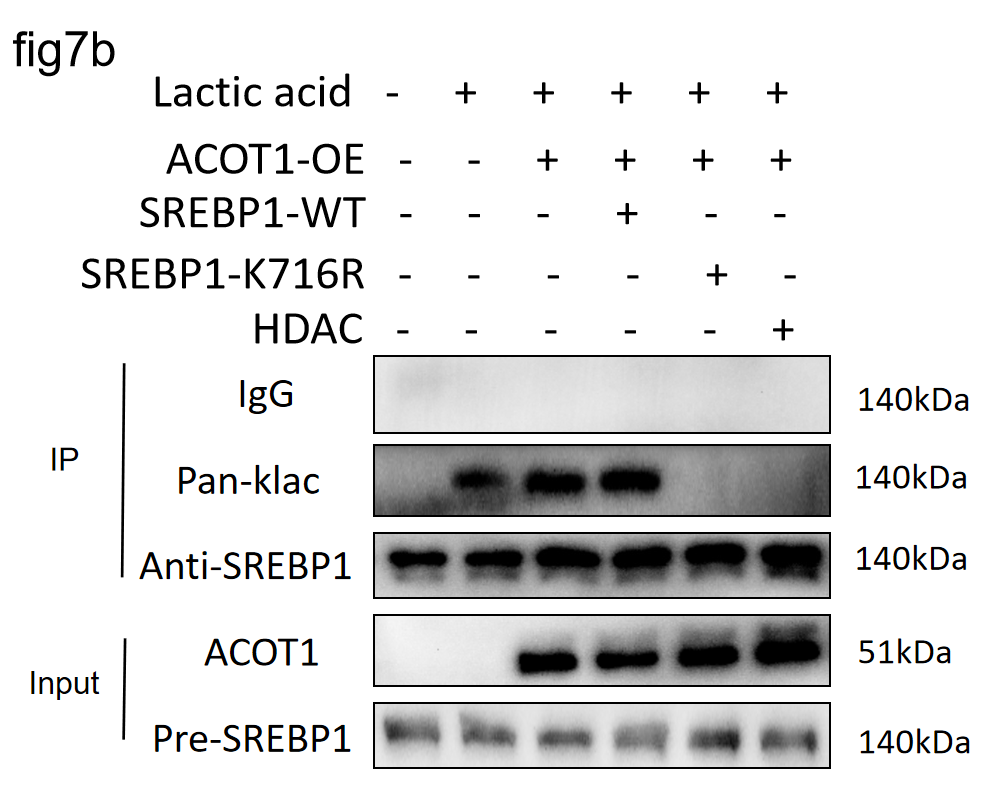

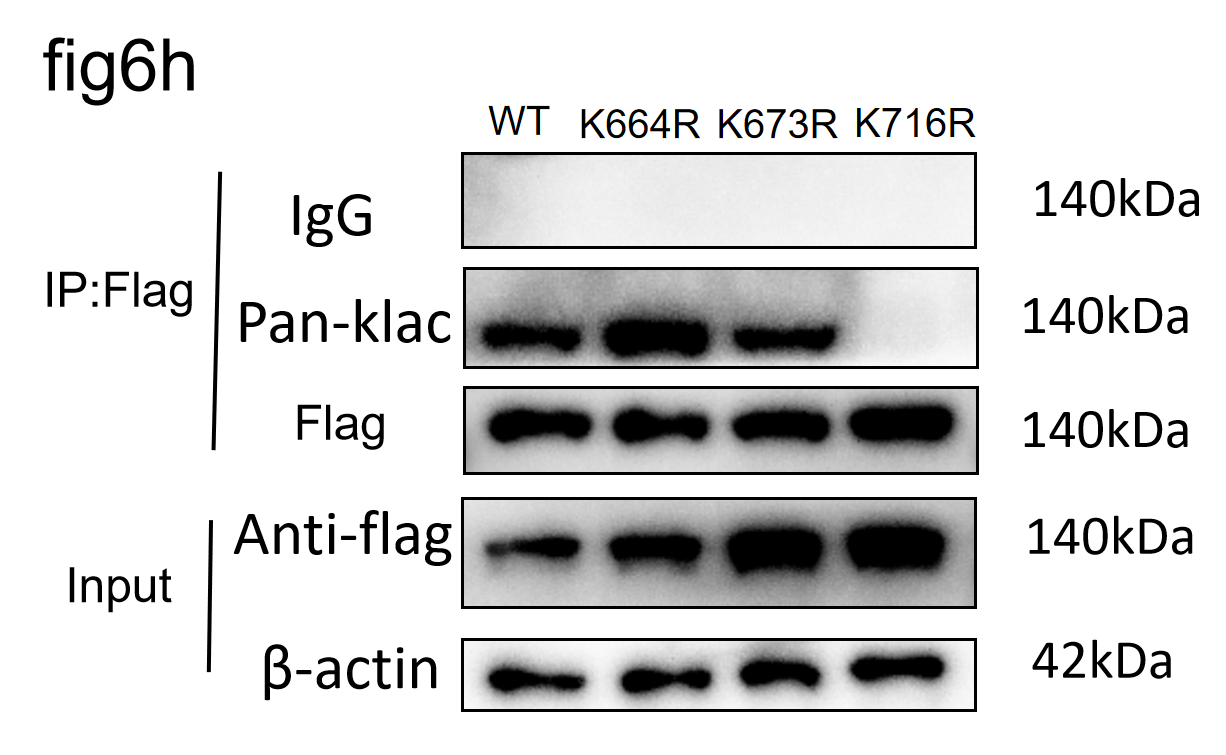

Supplement: Supplementary Fig. S3 — Western blot images with IgG controls for all Co-IP experiments. [file mmc3.docx]
