# Supplementary material for: ACOT1-specific expression modulates metabolic reprogramming in diabetic cardiomyopathy: The role of SREBP1c lactylation in CD36-mediated lipotoxicity
Source: Am Heart J Plus. 2026 Jun 6;67:100809. doi: 10.1016/j.ahjo.2026.100809 (PMC13260214; doi:10.1016/j.ahjo.2026.100809)
Supplement: Supplementary Fig. S4 — ACOT1 overexpression increases intracellular diacylglycerol (DAG) accumulation. [file mmc4.docx]

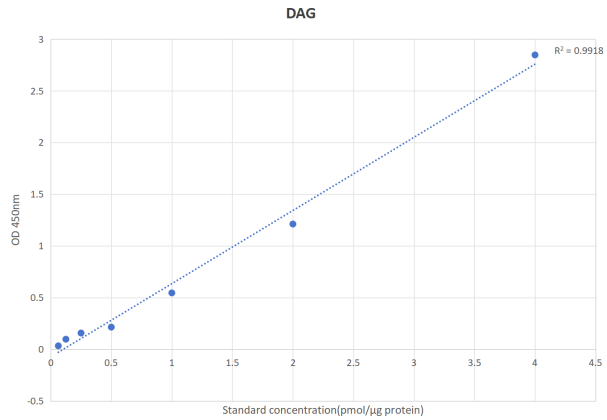

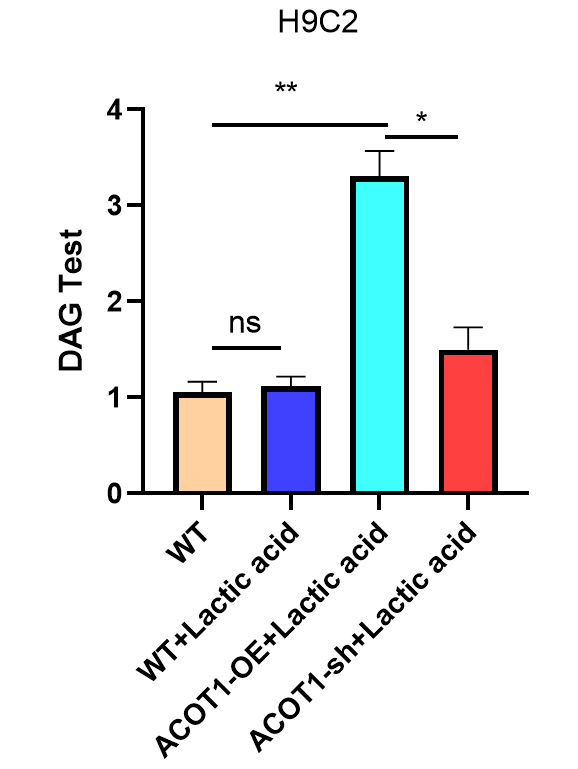


| Group | Mean ± SD (pmol/µg) | SEM |
| --- | --- | --- |
| WT | 1.054 ± 0.109 | 0.049 |
| WT + Lactic acid | 1.115 ± 0.101 | 0.045 |
| ACOT1-OE + Lactic acid | 3.309 ± 0.259 | 0.116 |
| ACOT1-sh + Lactic acid | 1.497 ± 0.235 | 0.105 |

Supplementary Figure S4. ACOT1 overexpression increases intracellular diacylglycerol (DAG) accumulation.

(a) Standard curve of the DAG ELISA assay. Absorbance (OD value) plotted against known DAG concentrations. The linear regression equation and R² value are indicated.

(b) Intracellular DAG levels in H9C2 cardiomyocytes. Cells were transfected with ACOT1 overexpression (OE) or knockdown (sh) constructs and treated with sodium lactate (20 mM, 24 h). DAG content was measured by ELISA and normalized to total protein. ACOT1 OE significantly increased DAG levels compared with all other groups, while ACOT1 knockdown reduced DAG to near-baseline levels. Data represent mean ± SEM; n = 5 independent biological replicates per group. One-way ANOVA with Tukey post-hoc test.
